# Staff perspectives on the implementation of interventions for people with congenital disabilities: a mixed-methods systematic review

**DOI:** 10.1186/s13643-026-03086-0

**Published:** 2026-02-02

**Authors:** Anette Granberg, Marie Matérne, Lars-Olov Lundqvist, Anna Duberg

**Affiliations:** 1 Faculty of Medicine and Health, University Health Care Research Centre, Örebro University, Örebro, Sweden; 2 School of Behavioural, Social and Legal Sciences, Örebro University, Örebro, Sweden; 3 Faculty of Medicine and Health, University Health Care Research Centre, Orebro University, S-huset, van 2, Orebro, SE-70185 Sweden

**Keywords:** Implementation, Barriers and facilitators, Strategies, Outcomes, Systematic review, Disability, Habilitation

## Abstract

**Background:**

The implementation of interventions in clinical practices is a challenge in healthcare settings, particularly in the field of habilitation. Although research on interventions in this area has increased, research on the implementation of these interventions has been slow. Exploring staff experience of implementation is therefore crucial to optimize the likelihood that interventions will be adopted and sustained in habilitation settings. The purpose of this systematic review is to synthesize the experiences of staff in implementing interventions for adults with congenital disabilities into a comprehensive overview.

**Methods:**

Studies were included if they provided empirical data on staff experiences of implementing interventions for adults with congenital disabilities in health and social care settings, regardless of study design. Non-empirical studies and grey literature were excluded. Following Preferred Reporting Items for Systematic Reviews and Meta-Analyses (PRISMA) methodology, we conducted a search using the Medline, CINAHL, PsycInfo, Sociological Abstract, Applied Social Sciences Index and Abstracts (ASSIA), and Web of Science databases. The last search update was conducted in February 2024. The Critical Appraisal Skills Program (CASP) tool was used for qualitative studies, and the Mixed-Method Assessment Tool (MMAT) for the quantitative and mixed-method studies. The Convergent Integrated Approach (CIA) was employed to synthesize the data.

**Results:**

Of the 5855 studies initially retrieved, eight met the inclusion criteria. The analysis and integration of all included studies were categorized into three themes: (1) conditions for implementation, (2) acceptability of the intervention, and (3) approaches for change. The results underscore the importance of organizational resources, vision, and collaboration in successful implementation.

**Conclusions:**

Active participation of professionals and alignment of interventions with existing practices were identified as key factors for success. The consideration of external factors that can influence the implementation of interventions is also important. In general, these findings provide information to guide future planning and implementation of interventions within habilitation settings.

**Systematic review registration:**

Open Science Framework (OSF): https://doi.org/10.17605/OSF.IO/SBV9E

**Supplementary Information:**

The online version contains supplementary material available at 10.1186/s13643-026-03086-0.

## Background

The implementation of interventions in clinical practice is challenging in healthcare settings [1, 2]. Habilitation, which focuses on supporting people with disabilities to develop and maintain functional abilities and participation [5], faces similar implementation challenges, including limited organizational resources, variability in staff training and competencies, and the need to tailor interventions to diverse client needs [6]. Crucially, the successful delivery of habilitation interventions depends on the actions, attitudes, and readiness of staff such as healthcare professionals and managers [7]. Staff are responsible for tailoring interventions, coordinating care across teams and sectors, and sustaining new practices over time. Despite the importance of intervention-based work in habilitation, little is known about how such interventions are implemented in everyday practice [7]. Broader rehabilitation research indicates that implementation processes are often under-studied [3], which may contribute to persistent gaps between available evidence and the care that patients ultimately receive [4].

Studies show that people with disabilities have unequal access to healthcare services, have higher unmet healthcare needs, and experience lower levels of health throughout their life compared with the general population [8]. Furthermore, people with congenital disability (a disorder that is present from birth) can be dependent on others for most of their life, depending on the severity of the underlying disability. In addition, these people often have problems communicating [9]; thus, they are dependent on the will, motivation, and attitude of staff during the implementation of interventions.

In general, interventions in the field of habilitation are recognized as more complex than other medical interventions [10]. This is because habilitation interventions involve a number of interacting components at the individual, practitioner, organizational, and system levels; they also need to be tailored to specific populations and contexts [11]. Habilitation services are located within complex multilevel healthcare systems involving interactions between individual healthcare professionals, multiprofessional teams, managers at different levels, patients, and their families and carers [8]. In this context, “the system” encompasses the wider habilitation and community-care structures—including specialist habilitation units, primary care, municipal services, and hospital wards. In addition, services may be provided by other professionals not specialized in habilitation outside the healthcare system, such as the municipality in public or private health settings [12]. Intervention implementations are therefore conducted in a wide range of settings that often involve different types of stakeholders, interventions, and implementation strategies [13–15]. A critical point in implementation is to find a fit between the new intervention and the context [16]. An implementation may require new routines for staff who may not be ready or even willing to embrace these changes [17]. Each setting may have its own unique context and challenges regarding factors that can inhibit or enable the implementation of an intervention, such as organizational support, financial resources, social relationships, leadership, and organizational culture and climate [18, 19]. Therefore, exploring the implementation experience of staff (e.g., healthcare professionals and managers) is crucial to optimize the likelihood that interventions will be adopted and sustained in habilitation settings [3, 18]. To improve access to interventions within habilitation services for people with disabilities, it is necessary to understand and explain what factors can affect the implementation of interventions by exploring barriers and facilitators, strategies, and implementation results in the specific context in which they are trialled [20–22].

The context is key and requires focus during implementation [22–25] because it may facilitate or constrain the implementation process [26]. Culture and leadership strongly shape whether an intervention succeeds because they influence priorities, communication, and staff readiness for change [24, 25, 27]. Clear and well-chosen implementation strategies—such as training, support, or workflow adjustments—can help staff adopt an intervention and continue using it in routine care [28–31]. In addition, research has shown that standardized measures and conceptual models for implementation outcomes are crucial to assessing the effectiveness of implementation strategies and promoting the dissemination of interventions. The literature also highlights the need for more robust theory building and empirical investigation to deepen the current understanding of implementation processes and their impacts [13, 32–35].

Research shows that habilitation faces several contextual barriers, such as limited resources and weak internal communication, which make it difficult to adapt interventions to patients’ needs [36, 37]. At the same time, studies indicate that habilitation requires tailored implementation strategies such as staff training, improved communication routines, and clearer team roles [17, 38, 39] because of its long-term and multidisciplinary nature [40–43].

However, there is still no comprehensive overview of which strategies are effective or how they should be applied in practice. This lack of synthesis makes it harder for professionals and managers to make informed decisions about how to support successful implementation. Educational meetings, materials, and outreach visits are commonly used, but standalone educational materials are typically insufficient to change the behavior of clinical practice behavior [44]. Although recent systematic reviews have focused mainly on barriers and strategies from the perspectives of patients, stakeholders, or caregivers [45–48], none have comprehensively synthesized the experiences of managers and healthcare professionals in multiple perspectives, such as barriers, facilitators, strategies, and outcomes, in the implementation of interventions for adults with congenital disabilities in healthcare settings. This systematic review aims to synthesize research on staff experiences on the implementation of interventions for adults with congenital disabilities.

## Methods

### Study design

We used a mixed methods review approach that included qualitative and quantitative data to identify, evaluate, and synthesize staff experience about the implementation of interventions [49]. The SPIDER (sample, phenomenon of interest, design, evaluation, and type of research) tool was used to structure the search terms and eligibility criteria [50]. This review followed the Preferred Reporting Items for Systematic Reviews and Meta-Analyses (PRISMA) [51]. The PRISMA checklist 2020 is provided in Additional file 2. The review protocol is provided in Additional file 3.

### Theoretical frameworks

The review is based on three key frameworks: the Integrated Promoting Action on Research Implementation in Health Services framework (i-PARIHS) [52, 53], the Cochrane Effective Practice and Organization of Care (EPOC) [54], and the Proctors’ taxonomy [32]. i-PARIHS is used for its ability to identify contextual factors that impede or enhance implementation processes and outcomes. The EPOC is used to further refine the identification of implementation strategies. The Proctors’ taxonomy is adopted for its focus on implementation outcomes distinct from service system and clinical treatment outcomes, providing valuable indicators for gauging implementation success [32].

### Search strategy

The search strategy was developed in consultation with medical health sciences librarians. Data was collected from peer-reviewed literature, with bibliographic databases as the main source. The medical health sciences librarians performed an electronic literature search on Medline, CINAHL, PsycInfo, Sociological Abstract, Applied Social Sciences Index and Abstracts (ASSIA), and Web of Science, as these databases were considered the most relevant and likely to cover the scope of this review. Details of the search strategies in the different databases are provided in the supplementary material (Additional file 4). The search was limited to articles published in English and Swedish, and no limitation was established on the publication date. The search terms, which included subject headings and free text terms, focused on identifying contextual factors, implementation methods and strategies, and implementation outcomes for interventions for people with congenital disabilities. However, the research team did not include staff (managers and healthcare professionals) in the search strategy because we tried to select widely and inclusively, indexing all available studies for the implementation of interventions. The last updated search was conducted in February 2024 (the initial search was made in March 2022 and was updated in August 2023).

### Eligibility criteria

Inclusion criteria were developed to guide the selection of studies. Any disagreement about eligibility was discussed and resolved in consensus within the research team.

#### Inclusion criteria

- *Sample:* staff (managers and healthcare professionals) working in hospital wards, habilitation services, nursing and residential homes, or community and private healthcare settings for adults (18 years or older) with congenital disabilities (i.e., a combination of long-term physical, intellectual, and sensory impairments that are present from birth or the first years). In cases where it was not possible to determine the specific type of disability of the patients with whom the staff worked, we chose to include the studies if all other criteria for inclusion were met. This approach was adopted to avoid excluding studies that included patients with various disabilities, even if those studies did not provide detailed descriptions of specific disabilities among patients.
- *Phenomenon of interest:* any studies reporting on
  (i) Staff experience of the influence of contextual factors on the implementation of interventions for people with congenital disabilities,
  (ii) Staff experience of techniques, methods, or strategies used to implement clinical interventions for people with congenital disabilities,
  (iii) Staff experience of implementation outcomes.
- *Study design:* any type of design
- *Evaluation:* quantitative and qualitative analysis of experiences, feelings, views, and opinions regarding implementation processes and not the effect of a clinical intervention.
- *Research type:* articles published in the English or Swedish language and based on empirical research reporting on primary data collected in practice settings

Thus, non-empirical studies such as systematic reviews, protocols, theoretical work, editorials, opinion pieces, and pilot studies, as well as conference presentations, book chapters, and dissertations, were not included.

### Study selection process

All identified studies were imported into reference management software (EndNote) and duplicate references were removed. The studies were then uploaded to Covidence [58], which is a platform that provides online systematic review management tools to manage the review process.

Firstly, two researchers independently of each other worked to screen the titles and abstracts of the studies according to the inclusion and exclusion criteria and to categorize each of them as (i) relevant, (ii) possibly relevant, or (iii) irrelevant. The full text of the studies classified as relevant or possibly relevant was then sifted through to determine whether the studies met the inclusion criteria. At this stage, the same two researchers independently selected studies that were eligible for final inclusion among full text studies. The entire research team then independently evaluated the full text of the included studies. Finally, two researchers independently reviewed the results of the full-text analysis, and the results were then discussed with the whole research team. Any discrepancies among the evaluations were resolved through a consensus discussion with the entire research team.

### Data extraction process

The research team developed a data extraction template using the Covidence tool to manage the data-extraction process [55]. First, two researchers planned, pilot tested, extracted data, and compared our template results until we reached a final decision on which data to collect. Then, the same two researchers independently worked on extracting data for all included studies. The data were then compared and discussed, and any discrepancies were resolved by consensus among the research team. For all included articles, we collected data comprising the authors, year of publication, title, study design, staff demographics, and intervention, as well as information on the staff’s experience of implementation of the intervention. The data extracted from the included studies are provided in the supplementary material (Additional file 5).

### Quality assessment

We used the Critical Appraisal Skills Program (CASP) tool for the qualitative studies [56] and the Mixed-Method Assessment Tool (MMAT) [60] for the quantitative and mixed-method studies. CASP was selected because it is a user-friendly method that enables systematic assessment of trustworthiness, relevance, and results of published papers for qualitative studies [56]. MMAT was selected because it can be used to assess quantitative and mixed methods research [57]. Two researchers independently reviewed all studies except one, using the CASP checklists or the MMAT critical appraisal tool. The quality assessment process involved a detailed review of the included studies to determine whether they met the criteria. For each criterion, the studies were marked as ‘Can’t tell’ (that is, it is unclear whether the study meets the criterion due to insufficient data or ambiguous reporting); ‘No’ (that is, clearly does not meet the criterion); or ‘Yes’ (that is, meets the criterion without reservation). In the cases where there was disagreement or uncertainty in the assessment, the researchers collaborated to reach a consensus through discussion. As the authors of the present paper wrote one of the included studies, an external researcher was invited to perform the quality assessment of that study.

### Synthesis of data

Nvivo software was used to manage the synthesis of the data and results [58]. The Convergent Integrated Approach (CIA) developed by Stern et al. [59] was employed to convert quantitative data into qualitative data, a process known as ‘qualitizing’. This approach facilitated the examination of staff experience in the implementation of interventions, incorporating both qualitative and quantitative data. Furthermore, the CIA helped determine whether the quantitative and qualitative data addressed various aspects of the phenomenon of interest. Using the method proposed by Frantzen et al. [60] quantitative data (QUAN) was transformed into qualitative data (QUAL) and integrated with qualitative categories and themes. This was done by following the steps below:

1. *Analyze and synthesize:* the QUAN and QUAL papers were synthesized with each other, according to the method described above. This approach ensured that a separate overview was created for both the QUAN and QUAL datasets. The synthesis was done by bringing the main findings from the respective datasets together, combining the results and interpretations by creating a new connected and summative whole covering all the QUAL and QUAN papers.
2. *Integrate:* this step involved bringing together the synthesized data (that had been transformed from QUAN to QUAL data and then synthesized) by constantly cross-checking, connecting, and co-informing each other. This process was done to ensure that the final synthesis was based on the results of both the QUAN and QUAL papers.
3. *Organize results:* the final synthesis was then organized and grouped into categories, while constantly trying to keep track of the results from each QUAN and QUAL paper that led to a category. Then, the concordance between the categories of the integrated datasets was examined.
4. *Draw conclusions:* finally, the categories and the overall integrated findings were produced by linking the results and categories into themes.

## Results

**Fig. 1:**
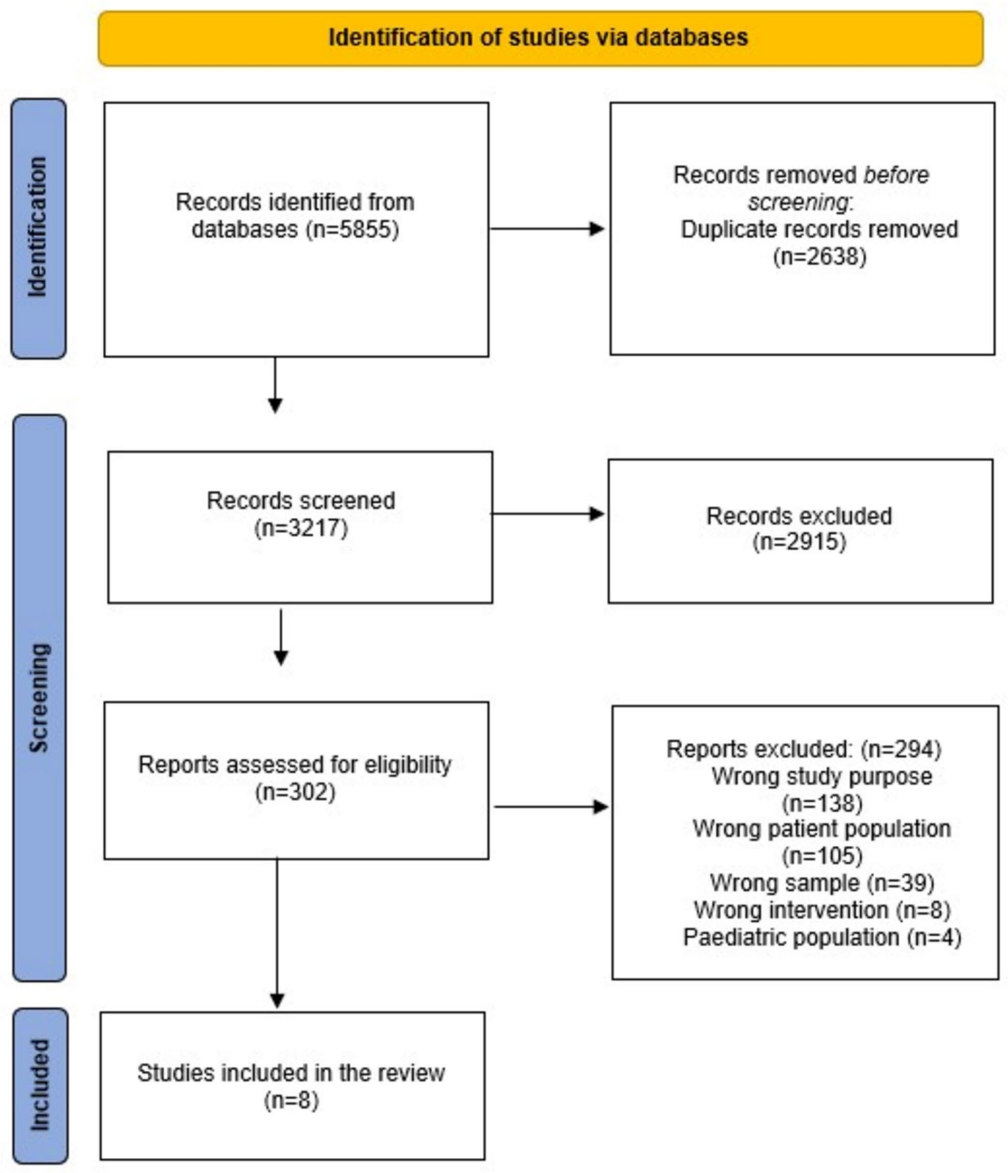
PRISMA flowchart of the study selection process

A total of 5855 studies were identified. Of these, 2638 were duplicates that were removed, resulting in 3217 studies. These studies were subjected to title and abstract screening, resulting in the exclusion of 2915 studies. Of the remaining 302 studies, a reading of the full texts against the inclusion and exclusion criteria resulted in eight studies being deemed relevant for this review. For a PRISMA flow chart of the study selection process, see Fig. 1.

The included studies were conducted in the Netherlands (*n* = 5), Sweden (*n* = 1), Canada (*n* = 1), and the USA (*n* = 1). They were conducted in rehabilitation services [61–64], the rehabilitation departments of hospitals [63, 64], habilitation services [65], a residential facility [66], a healthcare organization [67] and with rehabilitation professionals across research, educational, and clinical settings [68]. Three of the studies used a qualitative approach [63, 65, 67], three were quantitative [61, 62, 68] and two used a mixed method [64, 66]. The studies using a qualitative approach commonly used interview methods [63, 65, 67] those using a quantitative approach [61, 62, 68] commonly used questionnaires for data collection, and the studies using mixed methods used a combination of questionnaires, documentation, interviews, and logbooks [64, 66]. A complete overview of the characteristics of the included studies (authors, year, country, study design, title, study aim, settings, participants, and occupation, patient group, and age) is provided in Additional file 6.

Most studies focused on reporting barriers and facilitators first, then strategies, and finally the outcomes of implementation.

The most frequently reported barriers and facilitators concerned contextual factors at the organizational and local levels, including resources, structures, culture, and leadership. Recipient characteristics were the next most common, reflecting how staff knowledge, skills, values, and beliefs shape implementation. Innovation characteristics were mentioned least often and related to how the design and evidence base of a programme or intervention influence uptake. These aspects also highlight how knowledge is translated into practice and which organizational conditions support or hinder this process [53]. Based on EPOC [54], the strategies reported by the reviewed studies included clinical practice guidelines, continuous quality improvement, educational meetings, patient-mediated interventions, educational materials, educational outreach visits, reminders, funding, local opinion leaders, and interprofessional education. Furthermore, the reported implementation outcomes based on Proctor taxonomy [32] were adoption, sustainability, and fidelity.

Qualitative articles met most quality assessment criteria such as trustworthiness, relevance, and results. However, the criteria regarding whether the researchers critically examined their own role, potential bias during the formulation of the research questions, how the researchers responded to events during the study, and implications in the research design were not met at all. Similarly, the mixed-method articles met most of the criteria for study methodology and design, some criteria being rated as ‘Can’t tell’ due to insufficient information for a definitive assessment. All quantitative articles met the criteria, except for addressing non-response bias. Despite these considerations, the researchers decided to include all the studies finally after summarizing the overall quality of the included studies. This decision was based on the perceived value of the studies and the importance of considering a wide range of evidence in the analysis process. Details on the quality assessments for all included studies are provided in Table 1.

**Table 1:** Quality checklist criteria

Quality checklist criteria	Number of included studies that met this criteria (rating yes)
Critical Appraisal Skills Programme (CASP; qualitative)	*(N* = *3)*
1. Was there a clear statement of the aims of the research?	3/3
2. Is a qualitative methodology appropriate?	3/3
3. Was the research design appropriate to address the aims of the research?	2/3
4. Was the recruitment strategy appropriate to the aims of the research?	3/3
5. Was the data collected in a way that addressed the research issue?	3/3
6. Has the relationship between researcher and participants been adequately considered?	0/3
7. Have ethical issues been taken into consideration?	2/3
8. Was the data analysis sufficiently rigorous?	2/3
9. Is there a clear statement of findings?	3/3
10. How valuable is the research? (no rating)	Rating not indicated for this item
Mixed-Methods Appraisal Tool (MMAT; mixed methods)	*(N* = *2)*
Are there clear research questions?	2/2
Do the collected data allow to address the research questions?	2/2
1.1 Is the qualitative approach appropriate to answer the research question?	2/2
1.2 Are the qualitative data collection methods adequate to address the research question?	2/2
1.3 Are the findings adequately derived from the data?	1/2
1.4 Is the interpretation of results sufficiently substantiated by data?	1/2
1.5 Is there coherence between qualitative data sources, collection, analysis and interpretation?	1/2
4.1 Is the sampling strategy relevant to address the research question?	2/2
4.2 Is the sample representative of the target population?	2/2
4.3 Are the measurements appropriate?	0/2
4.4 Is the risk of nonresponse bias low?	2/2
4.5 Is the statistical analysis appropriate to answer the research question?	2/2
5.1 Is there an adequate rationale for using a mixed-methods design to address the research question?	2/2
5.2 Are the different components of the study effectively integrated to answer the research question?	2/2
5.3 Are the outputs of the integration of qualitative and quantitative components adequately interpreted?	2/2
5.4 Are divergences and inconsistencies between quantitative and qualitative results adequately addressed?	1/2
5.5 Do the different components of the study adhere to the quality criteria of each tradition of the methods involved?	**1/2**
Mixed-Methods Appraisal Tool (MMAT; quantitative descriptive)	*(N* = *3)*
Are there clear research questions?	3/3
Do the collected data allow to address the research questions?	3/3
4.1 Is the sampling strategy relevant to address the research questions?	3/3
4.2 Is the sample representative of the target population?	3/3
4.3 Are the measurements appropriate?	3/3
4.4 Is the risk of nonresponse bias low?	0/3
4.5 Is the statistical analysis appropriate to answer the research question?	3/3

### Key findings

The analysis and synthesis of all included articles revealed six categories related to the implementation of interventions, as outlined in Table 2. These categories were condensed into three overarching themes: (1) conditions for implementation, (2) acceptability of the intervention, and (3) approaches for change.

**Table 2:** Qualitative and qualitized data, with categories condensed into themes

Data and reference	Example of qualitative and qualitized data	Category and included studies (*n* = 8) number of citations within the category (ref =)	Themes
Qualitized data from mixed study [67]	Low routinization of intervention components leads to less result regarding the sustainability of the intervention	Organizational structure and culture (*N* = 8/ref = 40)	Conditions for implementation
Qualitized data from quantitative study [65]	The coordinators were able to create a culture in which rehabilitation professionals believed in the idea to integrate physical activity promotion in rehabilitation care		
Qualitized data from quantitative study [71]	According to the keyword analysis, the clinicians primarily felt that they lacked the time and educational resources necessary to learn/use the rehabilitation treatment specification system (RTSS)	Financial and personnel resources (*N* = 8/ref = 35)	
Qualitized data from quantitative study [65]	The financial incentives and advisory support were essential ingredients for a successful implementation		
Qualitative data [68]	Nothing was removed from the ordinary workload, so every new task added to the burden		
Qualitized data from mixed study [67]	The awareness and compatibility of a new intervention were moderate in stable, high-fidelity organizations. In improving-fidelity organizations, the awareness and compatibility were below average	Relevance of interventions (*N* = 5/ref = 16)	Acceptability of the intervention
Qualitized data from quantitative study [64]	The participants considered themselves ready to adopt e-health in their work, and they had a favorable view of the technologies in place. They perceived the organization as being only moderately ready for e-health changes. Perceived workload and position/duties in the organization were found to have an impact on readiness for e-health		
Qualitative data [66]	Setting up the centre was initiated by managers without any support from other professionals working in the department, which created resistance against the implementation plan	Support from professionals (*N* = 4/ref = 16)	
Qualitative data [68, 70]	To establish a change process, it is not enough to have a business plan. To reach effectiveness, communication is central to carry out the change and to understand why professionals should do it	Communication and collaboration (*N* = 5/ref = 12)	Approaches for change
Qualitative data [70]	The interviewees discussed that it would be helpful if all the professionals involved were aware of the aim of the intervention and of its importance		
Qualitized data from mixed study [69]	The intended dose, reach and fidelity relating to the implementation of a new intervention were not achieved. Despite this, the professionals understood the intervention; it also increased their engagement in existing activities and their ability to explore new ones	Professionals’ attitude and ability (*N* = 4/ref = 16)	
Qualitized data from quantitative study [64]	The older group differed from the younger respondents on the Individual and Technological subscales: the former saw themselves as less ready to use new technologies; they also had a less favorable view of the technologies currently used in the organization		

### Theme—conditions for implementation

The conditions for implementation theme included the categories’ *organizational structure and culture* and the *financial and personnel resources* in the implementation process; it outlined activities to achieve organizational goals for implementation and included rules, roles, and responsibilities. Furthermore, organizations that were able to create a culture based on a widely shared set of beliefs, values, and attitudes that were supported by strategies and structure for the implementation process appeared to be more successful in implementing interventions.

#### Organizational structure and culture

Barriers related to the organizational level (meso) centered on organizational unwillingness to adopt new interventions [63], lack of resources [64] and lack of overall organizational visions, goals, and structure to carry out the implementation of interventions [65]. Barriers associated with resources mainly involved a lack of financial and personnel resources to manage the implementation process [61, 63, 65, 67, 68].

Managers at higher levels in some organizations had views on what to prioritize when implementing new interventions or programmes [65]. For example, in some hospitals’ rehabilitation departments, managers did not recognize a new sport and exercise program as a key point of attention in their care. This resulted in uncertainty about the continuation of the program and future plans among the staff who provided this new program in the hospitals. Furthermore, although implementation strategies were used, some organizations had limited facilities for this type of sport and exercise program, so the program was not offered to people with disabilities [63].

One article [66] reported that the intended dose for the training program for staff on the implementation of an intervention was not achieved in the organizations studied. However, the study showed that the interventions were well executed and that the professionals’ understanding of the assignment, such as purpose, expectations, and how to apply the intervention in practice, was adequate [66]. However, another study [61] demonstrated less effective implementation outcomes, attributed to professionals grappling with conflicting guidance. Specifically, they encountered discrepancies between national guidelines and the vision of local organizations for health promotion when implementing new interventions [64]. The study indicated that smaller organizations tended to initiate the implementation of the intervention earlier than medium and large organizations. Furthermore, smaller organizations exhibited a more structured implementation approach compared to medium and large organizations [64]. Another key factor facilitating implementation fidelity within organizations was the presence of professionals engaged with clearly defined roles in the implementation process [64]. However, barriers related to organizational culture primarily revolved around staff behavior, such as resistance, lack of engagement, and lack of motivation for implementation. These barriers often occurred at the local level (micro) due to the lack of engagement and motivation [62, 63, 65], or disparities in cultures and interests between departments during the implementation of the intervention implementation [62].

One study [62] investigated the implementation of a new intervention using educational meetings as the key implementation strategy. It revealed that resistance of healthcare professionals to change and their lack of commitment to training led to less favorable outcomes in the implementation program, a finding consistent with other studies [62, 63, 65, 66]. On the contrary, organizations (meso-level) that developed a culture in which professionals integrated interventions into care exhibited committed and enthusiastic staff with positive attitudes [62, 63].

#### Financial and personal resources

Financial incentives, training programs, and internal and external advisory support were strategies considered essential ingredients for the sustainability of new interventions in practice [62]. A training program for staff (educational meetings) was considered a necessary strategy to reinforce new processes for implementation within organizations [63, 68]. These organizations often had an explicit vision and strategy for the implementation of interventions [63, 64], as well as preconditions such as leadership support and organizational structure to create a stimulating culture and realistic expectations regarding what implementation work could be performed [65]. However, staff workload, high staff turnover, and reorganizations resulted in a lack of time for implementation [61–63, 65, 66]. Due to a lack of time, researchers reported challenges in establishing and maintaining intersectoral collaborations both within the organization and outside the organization for implementation [62, 68]. Furthermore, perceived workload and position/duties in the organization were found to have an impact on the readiness of staff for change within the organization [61]. In addition, the scarce time and high costs for certain professions resulted in the low routinization of new interventions, leading to less promising results in terms of the sustainability of interventions in the longer term [64]. Managers struggled between balancing managerial duties versus leading change, resulting in a lack of time to work with implementation processes [65]. Additionally, staff expressed a lack of time for implementation processes because nothing was removed from their ordinary workload, so each new task added to their burden [63, 65]. Furthermore, the writing of project plans, annual plans, and reports for the implementation of interventions was described as time-consuming [63].

### Theme—acceptability of the intervention

This theme describes factors that are central to the *relevance of interventions*, such as knowledge, awareness of the purpose, the ability to adapt the intervention to regular practice, and that the intervention was perceived to be beneficial to patients. Additionally, *support from professionals and managers within the organization* (meso level) was found to be central to the success of the implementation process, as well as gaining access to materials and strategy support during the implementation process.

#### Relevance of interventions

Interventions that aligned with the existing practice and values of the organization were more likely to be reported as successful [63, 64]. The professionals were more likely to adopt a new intervention when it contained a clear content and provided added value [66], when they had the flexibility to adjust their existing daily work during implementation, and when outcomes on the patient level were visible to the involved professionals [62, 63]. Furthermore, the key to integrating a new intervention was related to the acceptance of the intervention [62]. Some professionals expressed that the implementation of new interventions provided new opportunities to improve patient participation, which increased the professionals’ motivation [63, 66]; however, they also raised questions about the necessary conditions and relevant implementation strategies for implementing new interventions [61]. The barriers included professionals being unaware of the objective of a new intervention and of its importance [67] and new interventions being difficult to understand [63, 68]. When an intervention was deemed unsuitable for patients, the professionals’ motivation was lower [63].

#### Support from professionals

Support from professionals [62–64] and managers [65] within the organizations was a key facilitator of the success of the implementation. Professionals’ appreciation and compatibility with the intervention were rated more positively by professionals (management, rehabilitation physicians, physiotherapists, sports therapists, and counsellors) working in organizations where implementation fidelity was high compared to organizations of moderate fidelity and improving-fidelity organizations [64]. Resistance among professionals was identified when the professionals were not actively involved in the implementation process [63, 67]. On the contrary, in organizations where professionals were actively involved, professionals with a proactive role were found to contribute positively to the implementation process [64]. Advisory support from the national level [62] or support from implementation programme coordinators [63] were other facilitating factors. Supporting strategies [62] and material for implementing and executing interventions were critical to implementation success [63]. Lack of support from managers [65] or other professionals (i.e., counsellor, physician, and project leaders) [63] could hamper the implementation process.

### Theme—approaches for change

This theme highlights *communication and collaboration* between departments, professionals, authorities, and other stakeholders outside the organization and describes how professionals perceived communication about decisions and the *attitude and ability of professionals* to work with implementation processes.

#### Communication and collaboration

The lack of communication and the lack of relationships between professionals, departments, and settings outside of healthcare was identified as a barrier to the implementation process [67]. Some professionals described a hierarchical relationship in which certain rehabilitation treatments were considered more important than others [63]. The lack of communication between government authorities, such as the social insurance agency, the municipality, and the regional administration, was found to act as a barrier [65]. However, improving communication and collaboration between professionals was a successful way to overcome these barriers [63]. Moreover, the researchers found that well-functioning communication and collaboration (in which ideas, thoughts, opinions, and knowledge are shared and understood with clarity and purpose) between the professionals in a multidisciplinary team during implementation processes appear to be essential strategies for successful implementation. Lack of communication regarding decisions can also be a barrier; for example, in one case, management did not communicate why it was mandatory for staff to participate in training programs for the implementation of interventions [66]. Another barrier described by professionals was when policy decisions were based on a lack of knowledge and understanding of patient needs and an unawareness of the negative consequences these decisions had for patient outcomes [65]. Collaboration between teams within and between organizations was identified as a major facilitating factor that could influence the implementation process [63, 65]. This was achieved when professionals shared common knowledge [67, 68] and skills to implement and execute interventions [63]. Sharing experiences with other professionals was another key facilitator, and collaboration outside the organization was expressed as a valuable contribution to implementation processes [63]. Problems such as poor relationships with other departments or organizations and an insufficient number of professionals involved were described as a key barrier to collaboration in one article and were found to hamper the implementation process [67].

#### Professionals’ attitude and ability

Staff who reported lower change skills generally expressed greater resistance to the implementation of interventions [61, 68]. However, the demands and expectations for staff knowledge and skills were high for implementation processes in organizations, where staff were generally more committed, motivated, and had the ability to see opportunities instead of problems with the implementation [65]. Facilitating factors included the knowledge and skills of managers for the implementation process and their ability to involve staff throughout the whole implementation process [65]. Identifying and training local opinion leaders seemed to be a successful strategy for the implementation of interventions [68]. Informal leaders could act as barriers or facilitators for the implementation process. For example, informal leaders could question the validity of the intervention; in contrast, in some cases, they were regarded as key players because they drove the implementation process forward [65].

## Discussion

The purpose of this mixed-methods systematic review was to synthesize research on staff experiences on the implementation of interventions for adults with congenital disabilities. This review highlights three themes: conditions for implementation, the acceptability of the intervention, and the approaches for change.

The theme *conditions for implementation* described the importance of resources, overall organizational visions, goals, structure, and routines for the implementation of interventions. Organizations that had a clear vision, goals, structure, and routines, and that supported the implementation process, succeeded more in intervention implementation in terms of acceptability and sustainability. These findings align with those of a systematic review conducted by Gifford et al. [69], which examined and statistically analyzed the relationship between managerial leadership behaviors and staff’s (i.e., nurses’ and allied health professionals’) utilization of research [69].

In addition, the organization’s ability to adopt new interventions has been frequently raised in implementation research to date; factors to consider in different settings and over time that are related to the organization’s ability include acceptability, fidelity, and feasibility (which is proposed to influence sustainability and scalability) during the preliminary phases of intervention development, evaluation, and implementation [15, 70].

The results of this review emphasize that professionals who had a clear role and were actively engaged and involved in the implementation process were key facilitators for implementation success. However, despite optimal conditions for organizational structure and supportive leadership, implementation can be undermined by changes outside the organization’s environment, such as fluctuation in funding, contracting practices, technology, legislation, clinical practice guidelines and recommendations, or other aspects of the organizational environment [71]. Consistent with this finding, the reviewed studies reported encountering differences between the implementation process according to national guidelines and the vision of the local organization in terms of promotion of health. The lack of communication and the lack of relationship with the settings outside the organization were found to be barriers to implementation. However, advisory support from the national level or support from implementation programme coordinators was found to facilitate the implementation process.

The theme *acceptability of the intervention* emphasized interventions that aligned with existing practice and values; furthermore, when the outcomes at the patient level were visible to the involved professionals, the interventions were more accepted and likely to be reported as successful. This result is largely consistent with the findings by Holmes et al. [21], who reported that ‘patient needs and resources’, ‘readiness for implementation’, ‘knowledge and beliefs about the intervention’, ‘facilitation strategies’, and ‘participant responsiveness’ were the most frequently reported barriers and facilitators. However, while much research in implementation science has focused on contextual factors, such as system and staff influences [22, 39, 72], recent research has emphasized that collaboration in intervention design plays an important role in implementation processes [15, 73]. Having an awareness of facilitators and barriers is especially important in the design and delivery of complex, multifaceted interventions within habilitation settings [41, 74]. Such awareness involves barrier identification, linking barriers to implementation strategies, use of theory, and user engagement (i.e., seeking input on the feasibility or acceptability of the intervention from the potential targets). In this review, we found that sharing the knowledge of professionals and being aware of the purpose of the implementation process were central.

The theme *approaches for change* highlighted challenges at the micro, meso, and macro levels, including the collaboration between departments/teams within and outside the organization, the impact of informal leaders, and the level of knowledge and skills. The most predominant strategies in the reviewed articles were educational-related ones, such as materials, meetings, and educating outreach visits. This finding is consistent with a systematic review by Jones et al. [75], who examined the strategies used and found that education-related strategies were the most predominant interventions [75, 76].

### Implications for staff and future research

The results of the reviewed studies offer valuable information on the factors that influence the implementation of interventions in habilitation settings for adults with congenital disabilities. To improve implementations and improve outcomes for this population, it is recommended to involve relevant stakeholders—including organizations, staff, and informal leaders—to ensure awareness of different relevant needs.

To advance current understanding and the application of effective interventions for adults with congenital disabilities, future research should prioritize the following areas.

- *Approaching the complexities of habilitation settings:* it is necessary to explore methods for navigating the intricate dynamics present in habilitation environments when designing and implementing new interventions/methods. This includes examining the unique challenges and opportunities that arise in habilitation settings, such as the need for individualized care and the importance of interdisciplinary collaboration.
- *Long-term sustainability of interventions:* it is necessary to investigate the durability of the interventions implemented and the factors that contribute to their lasting success. With a focus on the sustainability of interventions within habilitation settings, exploring these topics can help improve the implementation of habilitation, resulting in better outcomes for people with disabilities. By addressing the complexities of habilitation settings and ensuring the long-term sustainability of interventions, future research can contribute to the development of effective interventions that meet the unique needs of people with congenital disabilities.

### Strengths and limitations

Using a mixed-method review approach made it possible to capture the entire implementation process from the perspective of the staff and within different habilitation settings. This is particularly important in a field with few studies using different data methods. Furthermore, using the i-PARIHS, EPOC, and Proctors [32, 53, 54] theoretical frameworks provided structure and clarity for the findings. However, some limitations of this systematic review should be noted. As we chose to include only peer-reviewed research published in English and Swedish, we may have been unable to include some of the recent research in the field that is written in other languages. In addition, since we chose studies published in peer-reviewed journals, we may have missed some relevant theses, reports, or unpublished studies. Furthermore, despite our efforts to accurately represent the contribution of Swedish articles, no Swedish articles were found by the search strategy within the bibliographic databases. Finally, although this is not necessarily a limitation, the search yielded a study by the research team, which could not be evaluated during the initial round of assessment. However, we invited an external researcher to review the quality of the study, and after approval, it was taken into consideration along with the other studies. While assessing the ease or difficulty of implementing the described interventions may be intriguing to practitioners in the habilitation field, it falls beyond the scope of this review. Additionally, we have not considered the various professions that could be involved in the implementation process. We aim to capture a broad range of relevant studies, while recognizing that doing so could limit the applicability of the findings to specific disability groups and would require critical evaluation when interpreting the results. Each implementation scenario is different and comes with its own set of challenges. Therefore, it is crucial to avoid interpreting the results of this review as a universal ‘recipe’ for how interventions should be implemented.

## Conclusions

The systematic review of mixed methods of staff experiences on the implementation of interventions for adults with congenital disabilities revealed three themes: conditions for implementation, acceptability of the intervention, and approaches for change. These themes underscore the importance of organizational resources, visions, and collaboration in successful implementation. Active participation of professionals and alignment of interventions with existing practices were identified as key factors for success. However, changes outside the organization can affect how well the implementation of the intervention works. Overall, these findings provide insights for guiding future planning and implementation of interventions within habilitation settings.

## Supplementary Information

Additional file 1: The PRISMA 2020 for abstract.

Additional file 2: The PRISMA 2020.

Additional file 3: Protocol.

Additional file 4: Electronic searches.

Additional file 5: Data items collected for the included studies.

Additional file 6: Characteristics of the included studies.

## Data Availability

The datasets used and/or analyzed during the current study are available from the corresponding author on reasonable request.

## Abbreviations

ASSIA: Applied Social Sciences Index and Abstracts
EPOC: Cochrane Effective Practice and Organization of Care
I-PARIHS: Integrated Promoting Action on Research Implementation in Health Services
PRISMA: Preferred Reporting Items for Systematic Reviews and Meta-Analysis
