# Supplementary material for: Staff perspectives on the implementation of interventions for people with congenital disabilities: a mixed-methods systematic review
Source: Syst Rev. 2026 Feb 2;15:77. doi: 10.1186/s13643-026-03086-0 (PMC12983735; doi:10.1186/s13643-026-03086-0)
Supplement: Supplementary file 3 — Additional file 3: Protocol. [file 13643_2026_3086_MOESM3_ESM.pdf]

TITLE

Implementation of interventions for people with congenital disabilities from the staff perspective: a mixed-methods systematic review protocol of barriers, facilitators, strategies and outcomes

Authors

Anette Granberg[1], Marie Matérne[1,2], Lars-Olov Lundqvist[1], Anna Duberg[1]

[1] University Health Care Research Centre, Faculty of Medicine and Health, Örebro University, Örebro, Sweden.
[2] School of Law, Psychology and Social Work, Örebro University, Örebro, Sweden.

Corresponding author:
Anette Granberg
anette.granberg@oru.se
University Health Care Research Centre
Faculty of Medicine and Health
Örebro University
Universitetssjukhuset Örebro, S-huset, vån 2
Örebro, SE-70185
Sweden

Note: This systematic review protocol adheres to The PRISMA 2020 statement: Updated guidelines for reporting systematic reviews (1).

INTRODUCTION

Rationale

The problem

The implementation of interventions in clinical practices is a challenge across healthcare disciplines (2, 3). It is important for organizations to address implementation challenges in healthcare and other settings in order to understand what happens when an intervention, programme, method or service is "interwoven" with current services by a team (4). Contextual factors, which can be broadly defined as all intervening factors that may influence the implementation of an intervention (5), have been recognized as important to implementation success (5-8), since they can be used to explain how or why certain implementation results are achieved (4).

Habilitation services aim to enable people with disabilities to develop functions they have never had or to maintain optimal functioning in their everyday lives(9); thus, habilitation contrasts with rehabilitation, as the latter aims to restore skills and functioning a person has lost (10).

Habilitation services are provided by healthcare professionals and are conducted within complex multi-level healthcare systems, involving interactions between individual healthcare professionals, multi-profession teams, managers at different levels, patients, and their families and carers (10). Services for people with disability may also be provided by other professionals not specialized in habilitation, outside of a healthcare context, such as by the municipality in public or private healthcare settings (9). The implementation of interventions can therefore be conducted in a wide range of settings that often involve different types of stakeholders, interventions and implementation strategies (11-13). Each setting has its specific context and challenges regarding contextual factors that may influence the implementation of interventions (14), such as

organisational support, financial resources, social relations, leadership, and organisational culture and climate (15). Furthermore, the influence of these contextual factors can explain the variation in implementation success (16). Therefore, it is important to examine the possible influence of contextual factors (i.e. facilitators and barriers) in order to identify possible paths/strategies for better implementation success (17). Such an investigation is crucial to optimize the likelihood of interventions being adopted and sustained in habilitation services (17), which is why implementation research is important.

Implementation research

Implementation research is the scientific inquiry into questions concerning implementation – that is, the act of carrying an intention into effect; in health research, implementation can be for policies, programmes, individual practices or interventions (18).

Factors affecting an implementation have been described in numerous theoretical frameworks. Contextual factors are explicitly reflected in the framework of Promoting Action on Research Implementation in Health Services (PARIHS) (19). Contextual factors may have an influence on implementation processes at the micro (individual), meso (team, department) and/or macro (hospital) level (20). The Cochrane Effective Practice and Organisation of Care (EPOC) taxonomy provides reviewers with guidance on how to organise and classifying interventions, as well as how to identify relevant information that could be extracted, such as which implementation strategies are used (21).

Furthermore, it is important to conceptualize and evaluate implementation outcomes in order to know whether an intervention is successfully implemented or not. Proctor et al.'s (22) taxonomy of outcomes for implementation research can be used to distinguished implementation outcomes from service system and clinical treatment outcomes.

Research in the field

Randomized controlled trial (RCT) designs are increasingly being used in habilitation research, but the implementation of interventions in habilitation practices is slow (17). Healthcare professionals in this field are concerned with factors that may influence the implementation of an intervention, other than whether an intervention or therapy is effective (23). In general, interventions in habilitation are recognized as more complicated than other medical interventions (24). This is because habilitation interventions generally involve a number of interacting components – as well as various intervention target levels such as the individual, practitioner, organizational and/or system levels – and may be tailored to population and context (25). A habilitation intervention may possess complexity across all these levels (24).

In addition, managers' leadership is an important factor in a successful implementation. That is, their leadership strongly influences their staff's use of research evidence, while a lack of managers' leadership has been identified as a major barrier to implementation (26-29). However, despite optimal conditions regarding organizational structure and a supportive leadership, implementation can be undermined by changes outside the organization's environment, such as fluctuations in funding, contracting practices, technology, legislation, clinical practice guidelines and recommendations, or other aspects of the organizational environment (30).

In their systematic review, Gifford et al. (31) examined the association between leadership and research use. Their review showed that managers use a range of leadership practices involving change, relations and task-oriented behaviours to facilitate and support their staff's use of research evidence in clinical practice. Another systematic review by Goorts et al. (32) examined the effectiveness of implementation strategies for disseminating and implementing evidence-based

guidelines in an allied health context. This review only included RCTs and other systematic reviews. The results showed that multi-faceted strategies appear to be the most effective for improving knowledge and adherence to guidelines and evidence.

In addition, a systematic review by Holmes et al. (33) examined barriers and facilitators affecting the implementation of a trial of complex interventions for adults with neurological impairment. The results were mapped to the Consolidated Framework for Implementation Research (CFIR). "Patient needs and resources", "readiness for implementation", "knowledge and beliefs about the intervention", "facilitation strategies" and "participant responsiveness" were the most frequently reported barriers and facilitators. Aspects relating to the quality of the intervention delivery and to organisational/contextual factors were rarely reported in studies.

Finally, a systematic review by Jones et al. (34) examined the strategies used and identified the methodological approaches utilized in studies in a habilitation context. The included studies were articles that only evaluated interventions or strategies for implementation. The review showed that education-related strategies were the most predominant interventions.

To date, no studies have synthesized the literature about managers' and healthcare professionals' experience of the influence of contextual factors on implementation (at the micro, meso and macro level), the strategies used for implementation or the implementation outcomes for people with disabilities in regional, municipal or private healthcare settings. Knowledge about these factors and strategies can further advance the understanding of the whole implementation process, making it possible to better understand whether and how interventions work in different settings. These important factors and strategies may facilitate the future implementation of evidence-based interventions in healthcare settings for people with disability.

Objectives

The objective of this systematic review was to compile a comprehensive overview of staff (i.e. managers' and healthcare professionals') experiences with the implementation processes of interventions for people with disabilities. The review attempts to answer the following questions:

How do staff in disability healthcare experience:

1. … the influence of contextual factors in the implementation of interventions?
2. … the methods and strategies used when implementing interventions in practice?
3. … implementation outcomes?

METHODS

Design

To guide this systematic review, we use an experiential (qualitative) review approach, which focuses on analyzing human experience as part of a larger phenomenon (23). To assess the participants' experience of the phenomenon, we have opted to use a mixed-methods approach and to include quantitative data. The SPIDER tool (sample, phenomenon of interest, design, evaluation and research type) is used to structure the search terms and eligibility criteria (35).

Eligibility criteria

Inclusion criteria

- *Sample:* Staff (managers and healthcare professionals) working in hospital wards, habilitation centers, nursing and residential homes, and community and private healthcare settings for adult people with disabilities. People with disability include adults (18–65 years old) with a combination of long-term physical, intellectual and sensory impairments that are present from birth.
- *Phenomenon of interest:* Any studies that report on staff's experience:
  - (i) of the influence of contextual factors (barriers and facilitators) on the implementation of interventions for people with disability;
  - (ii) of techniques, methods or strategies used to implement interventions for people with disability;
  - (iii) of implementation outcomes.
- *Design:* Any design
- *Evaluation:* Quantitative and qualitative analyses of experiences, feelings, views and/or opinions.
- *Research type:* Articles based on empirical research (i.e. observation and measurement of phenomena) in which the conclusions of the study are drawn from empirical evidence will be included. To be considered an empirical research report, the article must be based on primary data collected in practice settings.

Exclusion criteria

- *Sample:* Staff (managers and healthcare professionals) working with children with disabilities or staff working with people who have acquired injuries.
- *Phenomenon of interest:* Studies that only examine the perspectives of patients.
- *Design:* No exclusion criteria.
- *Evaluation:* Studies that only examine the effect of an intervention.
- *Research type:* Systematic reviews, protocols, theoretical work, editorials, opinion pieces, pilot studies and conference posters not available in full text, book chapters, and dissertations; papers published in languages other than English.

Information sources

Data will be collected from peer-reviewed literature, with bibliographic databases as the main source, and will be collected from a variety of bibliographic databases, including Medline, CINAHL, PsycInfo, Sociological Abstract, ASSIA (Applied Social Sciences Index and Abstracts) and Web of Science.

Search strategy

We will develop our search strategy in consultation with medical health sciences librarians. We will include all published articles. The search terms will include subject headings and free-text terms, and focus on identifying contextual factors, implementation methods and strategies, and implementation outcomes.

Selection process

Search results will be stored in EndNote X9 and uploaded to Covidence (www.covidence.org), a Cochrane technology platform that provides online systematic review management tools.

First, two researchers (AD, AG) will screen the titles and abstracts of the studies independently from the identified search, by categorizing the studies as relevant, possibly relevant or irrelevant, according to the review questions. Studies that pass the title and abstract screening process will then be assessed for inclusion and exclusion criteria. The full text of the studies will be sifted through in

order to determine whether the studies meet the criteria. In this stage, two researchers (AG, AD) will select studies independently to determine their eligibility for the final inclusion of the full text studies. Any disagreement between the authors regarding eligibility will be resolved in consensus with the research team (AG, MM and AD). A flow diagram following the PRISMA guidelines for reporting systematic reviews will be used to illustrate the selection processes and results (1).

Data-collection process
The next step will be to identify and present information from each selected study in order to summarize the key characteristics of each study and obtain an overview of the results. Then, the research team will develop a data-extraction template provided by Covidence to determine which variables to extract in order to answer the review questions. As this step is considered an iterative process, the data-extraction template will be continually updated if necessary. We will extract both general information on each study and specific information related to review questions. Two review authors will independently assess the data extraction. The extraction will thenen be sent for consensus among the research team (AG, MM and AD).

Data items
The following data items will be collected for the studies included in this review:

- **Information about the article**
  (Author(s), year of publication, title, DOI, country)
- **Information about the study**
  (Purpose of the study, study design, participants)
- **Staff demographics**
  (Sex, workplace, occupation and other characteristics)
- **Intervention**
  (Quantity, dosage, route of administration, format, duration, time frame, setting)
- **Contextual factors**
  (Staff experience of the influence of barriers and facilitators on the implementation of interventions; studies reporting on any factors that may influence the implementation of an intervention. To address barriers and facilitators, strategies can be developed to increase the pace and effectiveness of implementation.)
- **Methods and/or strategies**
  (Staff experience of methods and/or strategies used for the implementation of interventions. Studies that report on any methods and/or strategies used to enhance the adoption, implementation and sustainability of an intervention.)
- **Implementation outcomes**
  (Staff experience of implementation outcomes. Studies that report on any “implementation outcomes” distinct from service system and clinical treatment outcomes, and are the effects of deliberate and purposive actions to implement new treatments, practices and services).

Quality assessment
Once the data-extraction stage of this review is complete, the next task is to assess the quality of the included studies. Two review authors will independently assess the quality and bias risk for the included studies by using the Critical Appraisal Skills Programme tool (CASP) (36). The CASP checklists

can be used for different research designs. The tool contains several questions, each of which focuses on a different methodological aspect of studies. Most checklists currently have three sections:
Are the results of the study valid? (Section A)
What are the results? (Section B)
Will the results help locally? (Section C)

These questions will help the research team to read and check the included studies for trustworthiness, results and relevance by classifying each study. We chose this tool because it is considered to be a user friendly, and the first author (AG) had no prior experience with formally appraising the quality of studies within systematic reviews.

Synthesis methods

First, we will provide an overall summary of the findings of the included studies. Then, we will perform a mixed-methods research synthesis, which allows us to examine staff experience (qualitative and quantitative evidence) of an implementation intervention to better understand whether and how an intervention works. It also gives us the opportunity to examine whether the quantitative and qualitative data address different aspects of a phenomenon of interest, which can assist in highlighting gaps in research.

The questions in this review can be answered by both quantitative and qualitative studies; therefore, we will follow a convergent, integrated approach to synthesise the findings. To direct this process, we use methodical guidance on mixed-methods systematic reviews (MMSRs) in the literature (37). Here, “convergent integrated” refers to a process of combining extracted data; it involves data transformation by converting quantitative data into qualitative data (i.e. qualitizing). The quantitative data in this review will be converted into themes and categories through the following steps:

1) Convert the quantitative data into declarative standalone sentences in a way that answers the review questions;
2) Assemble the textual descriptions from quantitative studies and pool them with the extracted qualitative data;
3) Identify categories on the basis of similarity in meaning;
4) If possible, aggregate catergories to produce the overall integrated findings, by linking the results and findings into a coherent whole (38).

REFERENCES

1. Page MJ, McKenzie JE, Bossuyt PM, Boutron I, Hoffmann TC, Mulrow CD, et al. The PRISMA 2020 statement: an updated guideline for reporting systematic reviews. BMJ 2021;372:n71.
2. Titler MG. The evidence for evidence-based practice implementation. Patient safety and quality: an evidence-based handbook for nurses. Rockville (MD): Agency Healthcare Res Qual (US); 2008.
3. Grol R, Grimshaw J. From best evidence to best practice: effective implementation of change in patients' care. Lancet 2003;362(9391):1225-30.
4. Nilsen P, Bernhardsson S. Context matters in implementation science: a scoping review of determinant frameworks that describe contextual determinants for implementation outcomes. BMC Health Serv Res 2019;19(1):189.
5. Ploeg J, Wong ST, Hassani K, Yous M-L, Fortin M, Kendall C, et al. Contextual factors influencing the implementation of innovations in community-based primary health care: the experience of 12 Canadian research teams. Prim Health Care Res Dev 2019;20.

6. Coles E, Wells M, Maxwell M, Harris FM, Anderson J, Gray NM, et al. The influence of contextual factors on healthcare quality improvement initiatives: what works, for whom and in what setting? Protocol for a realist review. Syst Rev 2017;6(1):1-10.
7. Davidoff F. Understanding contexts: how explanatory theories can help. Implement Sci 2019;14(1):23.
8. Dryden-Palmer KD, Parshuram CS, Berta WB. Context, complexity and process in the implementation of evidence-based innovation: a realist informed review. BMC Health Serv Res 2020;20(1):81.
9. Stucki G, Cieza A, Melvin J. The international classification of functioning, disability and health: A unifying model for the conceptual description of the rehabilitation strategy. J Rehabil Med 2007;39(4):279-85.
10. World Health Organisation. WHO global disability action plan 2014-2021: better health for all people with disability. Geneva: World Health Organisation;2015.
11. Powell BJ, Fernandez ME, Williams NJ, Aarons GA, Beidas RS, Lewis CC, et al. Enhancing the impact of implementation strategies in healthcare: a research agenda. Public health Front 2019:3.
12. Vinson, Cynthia A., Katherine A. Stamatakis, and Jon F. Kerner. Dissemination and implementation research in community and public health settings. In: Ross C. Brownson, Graham A. Colditz, Enola K. Proctor (eds). Dissemination and Implementation Research in Health*:* Translating Science to Practice. New York: Oxford Academic 2017:355-70.

13. Klaic M, Kapp S, Hudson P, Chapman W, Denehy L, Story D, et al. Implementability of healthcare interventions: an overview of reviews and development of a conceptual framework. Implement Sci 2022;17(1):10.
14. Damschroder LJ, Aron DC, Keith RE, Kirsh SR, Alexander JA, Lowery JC. Fostering implementation of health services research findings into practice: a consolidated framework for advancing implementation science. Implement Sci 2009;4(1):50.
15. Rogers L, De Brún A, McAuliffe E. Defining and assessing context in healthcare implementation studies: a systematic review. BMC Health Serv Res 2020;20(1):591.
16. Pfadenhauer LM, Gerhardus A, Mozygemba K, Lysdahl KB, Booth A, Hofmann B, et al. Making sense of complexity in context and implementation: the Context and Implementation of Complex Interventions (CICI) framework. Implement Sci 2017;12(1):21.
17. Morris JH, Bernhardsson S, Bird M-L, Connell L, Lynch E, Jarvis K, et al. Implementation in rehabilitation: a roadmap for practitioners and researchers. Disabil Rehabil 2020;42(22):3265-74.
18. Peters DH, Adam T, Alonge O, Agyepong IA, Tran N. Implementation research: what it is and how to do it. BMJ 2013;347:f6753.
19. Harvey G. Implementing evidence-based practice in healthcare: a facilitation guide. London: Routledge/Taylor & Francis Group 2015.
20. Harvey G, Kitson A. PARIHS revisited: from heuristic to integrated framework for the successful implementation of knowledge into practice. Implement sci 2015;11(1):1-13.
21. Effective Practice and Organisation of Care (EPOC). EPOC Taxonomy [online]; 2015 epoc.cochrane.org/epoc-taxonomy (accessed 26 January 2022).
22. Proctor E, Silmere H, Raghavan R, Hovmand P, Aarons G, Bunger A, et al. Outcomes for implementation research: conceptual distinctions, measurement challenges, and research agenda. Admin Policy Ment Health 2011;38(2):65-76.
23. Munn Z, Peters MDJ, Stern C, Tufanaru C, McArthur A, Aromataris E. Systematic review or scoping review? Guidance for authors when choosing between a systematic or scoping review approach. BMC Med Res Methodol 2018;18(1):143.
24. Kayes NM, Martin RA, Bright FA, Kersten P, Pollock A. Optimizing the real-world impact of rehabilitation reviews: increasing the relevance and usability of systematic reviews in rehabilitation. Eur J Phys Rehabil Med 2019;55(3):331-41.

25. Craig P, Dieppe P, Macintyre S, Michie S, Nazareth I, Petticrew M. Developing and evaluating complex interventions: the new Medical Research Council guidance. BMJ 2008;337.
26. Al-Abri R. Managing change in healthcare. Oman Med J 2007;22(3):9-10.
27. Azad N, Anderson HG, Jr., Brooks A, Garza O, O'Neil C, Stutz MM, et al. Leadership and management are one and the same. Am J Pharm Educ 2017;81(6):102-.
28. Gifford WA, Squires JE, Angus DE, Ashley LA, Brosseau L, Craik JM, et al. Managerial leadership for research use in nursing and allied health care professions: a systematic review. Implement Sci 2018;13(1):127-.
29. Granberg A, Matérne M, Lundqvist L-O, Duberg A. Navigating change – managers' experience of implementation processes in disability health care: a qualitative study. BMC Health Serv Res 2021;21(1):571.
30. Haines ER, Birken SA. Organizational perspectives in implementation science. In: Per. Nilsen, Sarah A. Birken (eds). Handbook on Implementation Science: Edward Elgar Publishing 2020.
31. Gifford WA, Squires JE, Angus DE, Ashley LA, Brosseau L, Craik JM, et al. Managerial leadership for research use in nursing and allied health care professions: a systematic review. Implement Sci 2018;13(1):1-23.
32. Goorts K, Dizon J, Milanese S. The effectiveness of implementation strategies for promoting evidence informed interventions in allied healthcare: a systematic review. BMC Health Serv Res 2021;21(1):1-11.
33. Holmes JA, Logan P, Morris R, Radford K. Factors affecting the delivery of complex rehabilitation interventions in research with neurologically impaired adults: a systematic review. Syst Rev 2020;9(1):1-17.
34. Jones CA, Roop SC, Pohar SL, Albrecht L, Scott SD. Translating knowledge in rehabilitation: systematic review. Phys Ther 2015;95(4):663-77.
35. Cooke A, Smith D, Booth A. Beyond PICO: the SPIDER tool for qualitative evidence synthesis. Qual Health Res. 2012;22(10):1435-43.
36. Programme. CAS. CRASP checklist [online] 2022 https://casp-uk.net/casp-tools-checklists/ (accessed May 2022).
37. Stern C, Lizarondo L, Carrier J, Godfrey C, Rieger K, Salmond S, et al. Methodological guidance for the conduct of mixed methods systematic reviews. JBI Evid Synth 2020;18(10).
38. Sandelowski M, Voils CI, Barroso J. Defining and designing mixed research synthesis studies. Res Sch 2006;13(1):29.
