# Supplementary material for: Staff perspectives on the implementation of interventions for people with congenital disabilities: a mixed-methods systematic review
Source: Syst Rev. 2026 Feb 2;15:77. doi: 10.1186/s13643-026-03086-0 (PMC12983735; doi:10.1186/s13643-026-03086-0)
Supplement: Supplementary file 4 — Additional file 4: Electronic searches. [file 13643_2026_3086_MOESM4_ESM.pdf]

Sociology Collection (Combines abstracts and references from the ASSIA and Sociological Abstracts databases) via Proquest

Last updated search date 2024-02-29 (initial search was made in March 2022 and was updated in August 2023)

Search terms			Results
Disability			
	1	(MAINSUBJECT.EXACT("Disability studies") OR MAINSUBJECT.EXACT("Rehabilitation")) OR ti("disability studies" OR "disability research" OR habilitation OR rehabilitation OR "disability health care" OR "disability studies" OR "disability research" OR habilitation OR rehabilitation OR "disability health care") OR ab("disability studies" OR "disability research" OR habilitation OR rehabilitation OR "disability health care")	
Context			
	2	ti(context OR contextual OR influence OR factor* OR setting* OR environment* OR barrier* OR facilitator* OR "system characteristics" OR "organizational driver" OR "organizational drivers") OR ab(context OR contextual OR influence OR factor* OR setting* OR environment* OR barrier* OR facilitator* OR "system characteristics" OR "organizational driver" OR "organizational drivers")	
Implementation			
	3	((MAINSUBJECT.EXACT("Implementation") OR MAINSUBJECT.EXACT("Program Implementation") OR MAINSUBJECT.EXACT("Policy Implementation")) OR MAINSUBJECT.EXACT("Adoption of Innovations") OR MAINSUBJECT.EXACT("Innovations") OR MAINSUBJECT.EXACT("Diffusion of innovation theory") OR MAINSUBJECT.EXACT("Health Planning")) OR (ti(implementability OR implementable OR implementation* OR implemention) OR ab(implementability OR implementable OR implementation* OR implemention) OR ti((integration NEAR/3 innovation) OR (adaptation NEAR/3 innovation ") OR (diffusion NEAR/3 innovation)")) OR ab((integration NEAR/3 innovation) OR (adaptation NEAR/3 innovation ") OR (diffusion NEAR/3 innovation)")))	
Intervention			
	4	(MAINSUBJECT.EXACT("Adoption of Innovations") OR MAINSUBJECT.EXACT("Diffusion of innovation theory") OR MAINSUBJECT.EXACT("Intervention")) OR (ti(intervention* OR (integration NEAR/3 innovation) OR (adaptation NEAR/3 innovation) OR (diffusion NEAR/3 innovation)) OR ab(intervention* OR (integration NEAR/3 innovation) OR (adaptation NEAR/3 innovation) OR (diffusion NEAR/3 innovation)))	
Combined sets			
	5	1 AND 2 AND 3 AND 4	
Limits: English, Swedish			
	6	5	279

Cinahl via Ebsco

Last updated search date 2024-02-29 (initial search was made in March 2022 and was updated in August 2023)

Search terms			Results
Disability			
	1	(MH "Rehabilitation") OR (MH "Health Services for Persons with Disabilities")	
	2	TI (“disability stud*” OR "disability research" OR habilitation OR rehabilitation OR “disability health care”) OR AB (“disability stud*” OR "disability research" OR habilitation OR rehabilitation OR “disability health care”)	
	3	1 OR 2	
Context			
	4	TI (context or contextual or influence or factor* or setting* or environment* or barrier* or facilitator* or "system characteristics" or "organizational driver*") OR AB (context or contextual or influence or factor* or setting* or environment* or barrier* or facilitator* or "system characteristics" or "organizational driver*")	
Implementation			
	5	(MH "Diffusion of Innovation") OR (MH "Implementation Science") OR (MH "Health and Welfare Planning")	
	6	TI (implementability OR implementable OR implementation* OR implemention) OR AB (implementability OR implementable OR implementation* OR implemention) OR TI ((diffusion N3 innovation) OR (integration N3 innovation) OR (adaptation N3 innovation) OR AB ((diffusion N3 innovation) OR (integration N3 innovation) OR (adaptation N3 innovation))	
	7	5 OR 6	
Intervention			
	8	(MH "Diffusion of Innovation")	
	9	TI intervention* OR AB intervention* OR TI (diffusion N3 innovation OR integration N3 innovation OR adaptation N3 innovation) OR AB (diffusion N3 innovation OR integration N3 innovation OR adaptation N3 innovation)	
	10	8 OR 9	
Combined sets			
	11	3 AND 4 AND 7 AND 10	
Limits: English, Swedish			
	12	11	948

PsycInfo via Ebsco

Last updated search date 2024-02-29 (initial search was made in March 2022 and was updated in August 2023)

Search terms	Results
Disability	

	1	(DE "Rehabilitation")	
	2	TI (“disability stud*” OR "disability research" OR habilitation OR rehabilitation OR “disability health care”) OR AB (“disability stud*” OR "disability research" OR habilitation OR rehabilitation OR “disability health care”)	
	3	1 OR 2	
Context			
	4	TI (context or contextual or influence or factor* or setting* or environment* or barrier* or facilitator* or "system characteristics" or "organizational driver*") OR AB (context or contextual or influence or factor* or setting* or environment* or barrier* or facilitator* or "system characteristics" or "organizational driver*")	
Implementation			
	5	(DE "Innovation")	
	6	TI (implementability OR implementable OR implementation* OR implemention) OR AB (implementability OR implementable OR implementation* OR implemention) OR TI ((diffusion N3 innovation) OR (integration N3 innovation) OR (adaptation N3 innovation)) OR AB ((diffusion N3 innovation) OR (integration N3 innovation) OR (adaptation N3 innovation))	
	7	5 OR 6	
Intervention			
	8	TI (intervention*) OR AB (intervention*) OR TI ((diffusion N3 innovation) OR (integration N3 innovation) OR (adaptation N3 innovation)) OR AB ((diffusion N3 innovation) OR (integration N3 innovation) OR (adaptation N3 innovation))	
Combined sets			
	9	3 AND 4 AND 7 AND 10	
Limits: English, Swedish			
	10	9	638

Database(s): Ovid MEDLINE(R) ALL 1946 to August 17, 2023

Last updated search date 2024-02-29 (initial search was made in March 2022 and was updated in August 2023)

Search terms			Results
Disability			
	1	Disability Studies/	
	2	Rehabilitation/	
	3	Health Services for Persons with Disabilities/	
	4	("disability stud*" or habilitation or rehabilitation or "disability health care" or "disability research").ab,kf,ti.	
	5	1 or 2 or 3 or 4	
Context			
	6	(context or contextual or influence or factor* or setting* or environment* or barrier* or facilitator* or "system characteristics" or "organizational driver*").ab,kf,ti.	

Implementation			
	7	implementation science/	
	8	diffusion of innovation/	
	9	Health Plan Implementation/	
	10	(implementability or implementable or implementation* or implemention or (diffusion adj4 innovation) or (integration adj4 innovation) or (adaptation adj4 innovation)).ab,kf,ti.	
	11	7 or 8 or 9 or 10	
Intervention			
	12	diffusion of innovation/	
	13	(intervention* or (diffusion adj4 innovation) or (integration adj4 innovation) or (adaptation adj4 innovation)).ab,kf,ti.	
	14	12 or 13	
Combined sets			
	15	5 and 6 and 11 and 14	
Limits			
	16	limit 15 to (english or swedish)	1629

Web of Science

Indexes=SCI-EXPANDED, SSCI, A&HCI, CPCI-S, CPCI-SSH, ESCI Timespan=All years

Last updated search date 2024-03-01 (initial search was made in March 2022 and was updated in August 2023)

Search terms			Results
Disability			
	1	TS=(("disability stud*" OR "habilitation" OR "rehabilitation" OR "disability health care" OR "disability research"))	
Context			
	2	TS=(("context" OR "contextual" OR "influence" OR "factor*" OR "setting*" OR "environment*" OR "barrier*" OR "facilitator*" OR "system characteristics" OR "organizational driver" OR "organizational drivers"))	
Implementation			
	3	TS=(("implementability" OR "implementable" OR "implementation*" OR "implemention" OR ("diffusion" NEAR/3 "innovation") OR ("integration" NEAR/3 "innovation") OR ("adaptation" NEAR/3 "innovation")))	
Intervention			
	4	TS=("intervention*" OR ("integration" NEAR/3 "innovation") OR ("adaptation" NEAR/3 "innovation") OR ("diffusion" NEAR/3 "innovation"))	
Combined sets			
	5	1 AND 2 AND 3 AND 4	
Limits: English, Swedish			
	6	1 AND 2 AND 3 AND 4	2361
