# Supplementary material for: Staff perspectives on the implementation of interventions for people with congenital disabilities: a mixed-methods systematic review
Source: Syst Rev. 2026 Feb 2;15:77. doi: 10.1186/s13643-026-03086-0 (PMC12983735; doi:10.1186/s13643-026-03086-0)
Supplement: Supplementary file 5 — Additional file 5: Data items collected for the included studies. [file 13643_2026_3086_MOESM5_ESM.pdf]

Additional file – Data items collected for the included studies in this review:

- **Information about the article**
  (Author(s), year of publication, title, DOI, country)
- **Information about the study**
  (Purpose of the study, study design, participants)
- **Staff demographics**
  (Sex, workplace, occupation and other characteristics)
- **Intervention**
  (Quantity, dosage, route of administration, format, duration, time frame, setting)
- **Contextual factors**
  (Staff experience of the influence of barriers and facilitators on the implementation of interventions. Studies that report on any factors, which may influence the implementation of an intervention. To address barriers and facilitators, strategies can be developed to increase the pace and effectiveness of implementation.)
- **Methods and/or strategies**
  (Staff experience of methods and/or strategies used for the implementation of interventions. Studies that report on any methods and/or strategies used to enhance the adoption, implementation, and sustainability of an intervention.)
- **Implementation outcomes**
  (Staff experience of implementation outcomes. Studies that report on any "implementation outcomes" distinct from service system and clinical treatment outcomes, and are the effects of deliberate and purposive actions to implement new treatments, practices and services.)
