# Supplementary material for: Staff perspectives on the implementation of interventions for people with congenital disabilities: a mixed-methods systematic review
Source: Syst Rev. 2026 Feb 2;15:77. doi: 10.1186/s13643-026-03086-0 (PMC12983735; doi:10.1186/s13643-026-03086-0)
Supplement: Supplementary file 6 — Additional file 6: Characteristics of the included studies. [file 13643_2026_3086_MOESM6_ESM.pdf]

Characteristics of the included studies (n=8)

Author(s), year, country (ref. no.)	Study design	Title	Aim of studies	Settings	Participants and occupation	Patient group and age
Granberg et al. 2021 (63) Sweden	Qualitative (semi-structured individual interview)	Navigating change – managers' experience of implementation processes in disability health care: a qualitative study	To explore managers' experience of the implementation process when transferring new practices into disability healthcare settings	Habilitation centres at 4 regions in central Sweden	23 managers with formal managerial responsibility for implementation processes from public and private healthcare for people with PIMD	Adults with profound intellectual and multiple disabilities (PIMD)
Hanzen et al. 2020 (64) Netherlands	Mixed methods (questionnaires, assignments, documentation, interviews, logbook)	Improving the participation of adults with visual and severe or profound intellectual disabilities: a process evaluation of a new intervention	To conduct a process evaluation to observe the implementation phase of "Care for participation+" (CFP+) within a residential facility	A residential care facility in the Netherlands	A total of 16 direct support professionals (DSP) participating in the process evaluation	Adults with visual and severe or profound intellectual disabilities (VSPID)
Hoekstra et al. 2017 (61) Netherlands	Qualitative (semi-structured interviews)	Professionals' perceptions of factors affecting implementation and continuation of a physical activity promotion programme in rehabilitation: a qualitative study	To describe professionals' perceptions of factors that facilitate or hamper the implementation and continuation of a physical activity programme in rehabilitation	Professionals from 12 rehabilitation centres and rehabilitation departments of 6 hospital	A total of 22 interviews with rehabilitation professionals (n=28) involved as project leader (n=21) or counsellor (n=7)	Adults with physical disabilities
Hoekstra et al. 2017 (62) Netherlands	Mixed methods (online registration system, surveys, logbooks, interviews)	Implementing fidelity trajectories of a health promotion programme in multidisciplinary settings: managing tensions in rehabilitation care	To generate insight into heterogeneity in the implementation of fidelity trajectories of a health promotion programme in multidisciplinary settings and the relationship with changes in patients' health behaviour	17 locations consisting of 12 rehabilitation centres and 5 rehabilitation departments of hospital	Rehabilitation professionals (managers, physicians, project leaders, counsellors); survey collected at three time points (T0, T1 and T2); professionals' response rates to the three surveys was T0= 69 professionals, T1=59 professionals, T3= 66 professionals; 23 professionals participated in interviews	Adults with physical disabilities
Hoekstra et al. 2021 (60) Netherlands	Quantitative (survey)	The implementation of a physical activity counselling programme in rehabilitation care: findings from the ReSpAct study	To evaluate the implementation of a physical activity counselling programme in rehabilitation and to study heterogeneity in received counselling and investigate its association with changes in patients physical activity outcomes	18 rehabilitation institutions (12 rehabilitation centres and 6 rehabilitation departments of hospitals) across the Netherlands	A total of 70 professionals (managers, physicians, project leaders, counsellors)	Adults with disabilities

Steenbergen et al. 2019 (65) Netherlands	Qualitative (semi-structured interviews)	Examining determinants of lifestyle interventions targeting persons with intellectual disabilities supported by healthcare organizations: usability of the measurement instrument for determinants of innovations	To ascertain whether the Measurement Instrument for Determinants of Innovation (MIDI) is useful for objective evaluation implementation	Four healthcare organizations in the northern part of the Netherlands (supporting people with intellectual disabilities with various domains)	Four professionals who were responsible for lifestyle interventions	Adults with intellectual disabilities
Touré et al. 2012 (59) Canada	Quantitative (self-administered questionnaire)	Assessment of organizational readiness for e-health in a rehabilitation centre	To assess organizational readiness for e-health among the staff of an out-patient rehabilitation centre and to identify the personal characteristics of potential users that may have influenced readiness	Rehabilitation centre in Montreal, Canada	A total of 211 people, with 137 clinicians (physical educators, specialized educators, occupational therapist, physiotherapist, physician, speech therapist, psychologist, social workers and others), 28 managers and 46 non-clinicians	Adults with physical disabilities
Van Stan et al. 2023 (66) United states	Quantitative (a cross-sectional survey)	Rehabilitation treatment specification system: identifying barriers, facilitators, and strategies for implementation research, education, and clinical care	To explore rehabilitation professionals’ experiences and perspectives of barriers to and facilitators for implementing the Rehabilitation Treatment Specification System (RTSS) in research, education and clinical care	Rehabilitation professionals across research, educational and clinical settings	111 rehabilitation professionals – including speech-language pathologists, occupational therapists, physical therapists, physicians, psychologists, researchers and clinic directors	Patients within rehabilitation treatments
